# Loss of Side-to-Side Connections Affects the Relative Contributions of the Sodium and Calcium Current to Transverse Propagation Between Strands of Atrial Myocytes

**DOI:** 10.3389/fphys.2018.01212

**Published:** 2018-09-04

**Authors:** Jichao Zhao, Ulrich Schotten, Bruce Smaill, Sander Verheule

**Affiliations:** ^1^Auckland Bioengineering Institute, University of Auckland, Auckland, New Zealand; ^2^Department of Physiology, Maastricht University, Maastricht, Netherlands

**Keywords:** atrial fibrillation, transverse propagation, discontinuous conduction, sodium, calcium, fibrosis, structural remodeling

## Abstract

**Background:** Atrial fibrillation (AF) leads to a loss of transverse connections between myocyte strands that is associated with an increased complexity and stability of AF. We have explored the interaction between longitudinal and transverse coupling, and the relative contribution of the sodium (I_Na_) and calcium (I_Ca_) current to propagation, both in healthy tissue and under diseased conditions using computer simulations.

**Methods:** Two parallel strands of atrial myocytes were modeled (Courtemanche et al. ionic model). As a control condition, every single cell was connected both transversely and longitudinally. To simulate a loss of transverse connectivity, this number was reduced to 1 in 4, 8, 12, or 16 transversely. To study the interaction with longitudinal coupling, anisotropy ratios of 3, 9, 16, and 25:1 were used. All simulations were repeated for varying degrees of I_Na_ and I_Ca_ block and the transverse activation delay (TAD) between the paced and non-paced strands was calculated for all cases.

**Results:** The TAD was highly sensitive to the transverse connectivity, increasing from 1 ms at 1 in 1, to 25 ms at 1 in 4, and 100 ms at 1 in 12 connectivity. The TAD also increased when longitudinal coupling was increased. Both decreasing transverse connectivity and increasing longitudinal coupling enhanced the synchronicity of activation of the non-paced strand and increased the propensity for transverse conduction block. Even after long TADs, the action potential upstroke in the non-paced strand was still mainly dependent on the I_Na_. Nevertheless, I_Ca_ in the paced strand was essential to provide depolarizing current to the non-paced strand. Loss of transverse connections increased the sensitivity to both I_Na_ and I_Ca_ block. However, when longitudinal coupling was relatively high, transverse propagation was more sensitive to I_Ca_ block than to I_Na_ block.

**Conclusions:** Although transverse propagation depends on both I_Na_ and I_Ca_, their relative contribution, and sensitivity to channel blockade, depends on the distribution of transverse connections and the axial conductivity. This simple two-strand model helps to explain the nature of atrial discontinuous conduction during structural remodeling and provides an opportunity for more effective drug development.

## Introduction

Atrial fibrillation (AF)—the most common tachyarrhythmia in clinical practice—leads to remodeling of the atrial myocardium that promotes the maintenance of the arrhythmia (Schotten et al., 2011). Both the fast process of electrical remodeling (shortening of the action potential duration, occurring within 1–2 days) and the much slower process of structural remodeling (changes in cell and tissue structure, developing over months to years) contribute to AF stabilization (Schotten et al., 2011). Early in the disease process, sinus rhythm may be restored in patients by decreasing excitability (class I drugs, sodium channel blockers) (Crijns et al., 1988) or increasing action potential duration (class III drugs, potassium channel blockers) (Vos et al., 1998). However, when AF has been present for a longer time, both classes of drugs lose their efficacy, both in AF patients (Crijns et al., 1988; Vos et al., 1998) and in animal models (Eijsbouts et al., 2006; Verheule et al., 2010).

Whereas atrial myocytes form large intercalated discs with abundant gap junction channels at end-to-end connections, transverse connections between myocytes strands are more sparse (Spach and Dolber, 1986; Dolber and Spach, 1989). The structural changes—most notable endomysial fibrosis (also referred to as “interstitial fibrosis” and “microfibrosis”)—that results from aging (Spach and Dolber, 1986; Koura et al., 2002) or AF itself (Verheule et al., 2013) lead to a further loss of transverse connections. As a consequence, transverse propagation can become discontinuous, allowing reentrant conduction to take place in relatively small tissue areas (microreentry) (Spach and Josephson, 1994; Spach and Boineau, 1997).

**Figure 1:**
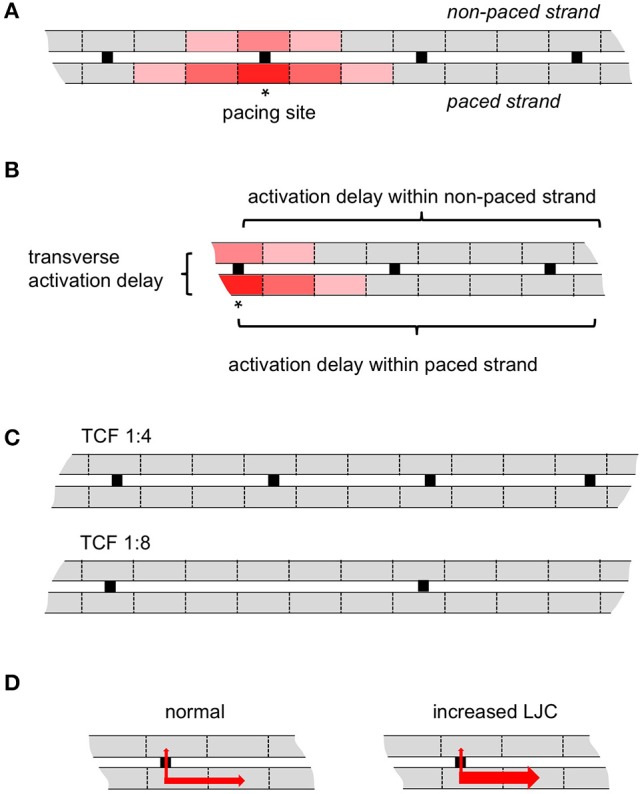
Schematic representation of the model. **(A)** Two parallel myocyte cables, in this case with a 1:4 discrete transverse connectivity ratio. Pacing (asterisk) was always delivered from middle of the lower strand (paced strand); the upper strand is non-paced strand. **(B)** The transverse activation delay is defined as the conduction time difference between the paced myocyte and the direct opposite myocyte in the non-paced strand; the activation delay within a strand is defined as the activation difference between 1st (stimulation site for paced strand or the direct opposite myocyte in non-paced strand) and 50th myocytes. **(C)** As an example, the transverse connectivity fraction (TCF) 1:4 and 1:8 are displayed here. **(D)** As an illustration for the longitudinal junctional conductance (LJC), the transverse conductivity is kept same but the longitudinal conductivity in both strands is increased.

In a seminal modeling study on longitudinal strands of ventricular myocytes, Shaw and Rudy have investigated the effects of reduced excitability (i.e., decreased sodium current) and gap junctional coupling (Shaw and Rudy, 1997). They showed that reduced sodium current amplitude led to a relatively small reduction in conduction velocity before conduction block occurred. By contrast, a reduction in gap junctional coupling allowed very slow conduction with a high safety factor (i.e., conduction that is unlikely to block). Under those conditions, electrical propagation became more dependent on the calcium current than on the rapidly inactivating sodium current. However, it has not been investigated how these ionic factors affect longitudinal and transverse propagation in strands of myocytes with transverse connections, and how structural remodeling (i.e., a loss of transverse connections) affects this interplay. We hypothesize that reduced transverse connectivity is a pivotal contributor to the substrate for sustained AF. Better understanding of the ionic basis of propagation can aid the development of more effective drug treatment for AF patients. Therefore, we have constructed a simple model consisting of two parallel strands of atrial myocytes with varying discrete side-to-side connections (Figure 1A). With this model, we have investigated the effect of reduced transverse connectivity on transverse propagation delays (Figure 1B), the relative roles of the sodium and calcium currents, and the sensitivity to sodium and calcium channel blockade.

## Methods

### Modeling atrial electrical transverse and longitudinal propagation in 3D cables

The two parallel myocyte cables with discrete transverse coupling were modeled in a 3D setting: 3 × 3000 × 3 cells in the upper strand, 3 × 3000 × 3 cells in the lower strand, transverse connections are composed as 3 × 1 × 3 cells (Figure 1). The spread of electrical activation in the 3D cable can be estimated by solving the cardiac monodomain equation (Zhao et al., 2013)

$$\begin{array}{l} \nabla •\left(\sigma_{i}\nabla {\text{V}}_{\text{m}}\right)={\text{A}}_{\text{m}}\left({\text{C}}_{\text{m}}\frac{\partial V_{\text{m}}}{\partial t}+{\text{I}}_{\text{ion}}\right) \end{array}$$

where the tensor σ_i_ represents the conductivity vector in spatial space, V_m_ is the transmembrane voltage, while A_m_ and C_m_ are membrane cross-sectional area and membrane capacitance. I_ion_ is the net current carried by transmembrane ion channels. The monodomain version of the reaction-diffusion equation was solved on a 3D voxel-based finite difference grid and paralleled using a Message Passing Interface (MPI). In the 3D cable model, we employed the biophysically detailed human atrial cell electrophysiology model developed by Courtemanche et al. (1998). Simulations with the Courtemanche cell model with a time step of 0.0025 ms run on an IBM3850 (32 dual thread Intel chips, 256 GB shared memory, Linux operating system) (Zhao et al., 2013). To simulate the loss of transverse connections in the cable model, we removed the connected grids in the grid-based mesh between the two parallel cables in the 3D numerical solver. Axially, anisotropic electrical properties were assigned to the two 3D cables along the long-axis of the cables and transversely in the cell-to-cell connection regions. Conductivities in transverse directions were fixed and set at 0.1 ms, while the longitudinal conductivities were varied to investigate the influence of longitudinal impedance. To model the effects of g_Na_ and g_Ca,L_ channel blockers in the 3D cable, we used the percentage of conductance g_Na_ and g_Ca,L_ of its baseline values.

### Computer simulation design

In this study, we have performed three groups of computer simulations:

1. Reducing discrete transverse coupling (1:1, 1:4, 1:8, 1:12, and 1:16) to study the impact of gradual loss of transverse connections during structural remodeling (Figure 1C);
2. Increasing the longitudinal conductance while keeping the transverse conductance the same (the ratio of the two at 3:1, 9:1, 16:1, and 25:1) to investigate the influence of longitudinal impedance on transverse propagation (Figure 1D);
3. Testing different degrees (0 to 100%) of sodium or calcium current blockade to investigate the sensitivity of longitudinal and transverse propagation to channel block.

In each of these simulations, the middle of the lower strand was paced. At that pacing site, 3 × 1 × 3 cells were paced with stimulus strength of 4 mA for 1 ms with 1 Hz. The results analyzed in this study were taken when the model reached its steady state (11th cycle). Atrial action potentials and sodium/calcium currents across the 3D myocyte cable were saved and analyzed, especially the site in the upper strand (the non-paced stand) that was directly opposed to the stimulus location (Figure 1B). A cut-off value of the membrane potential of −40 mV was used to determine whether a cell was activated or whether conduction had blocked. The time point with the maximal dV/dt was taken as the time point of activation.

## Results

### Effects of transverse connectivity on transverse propagation

**Figure 2:**
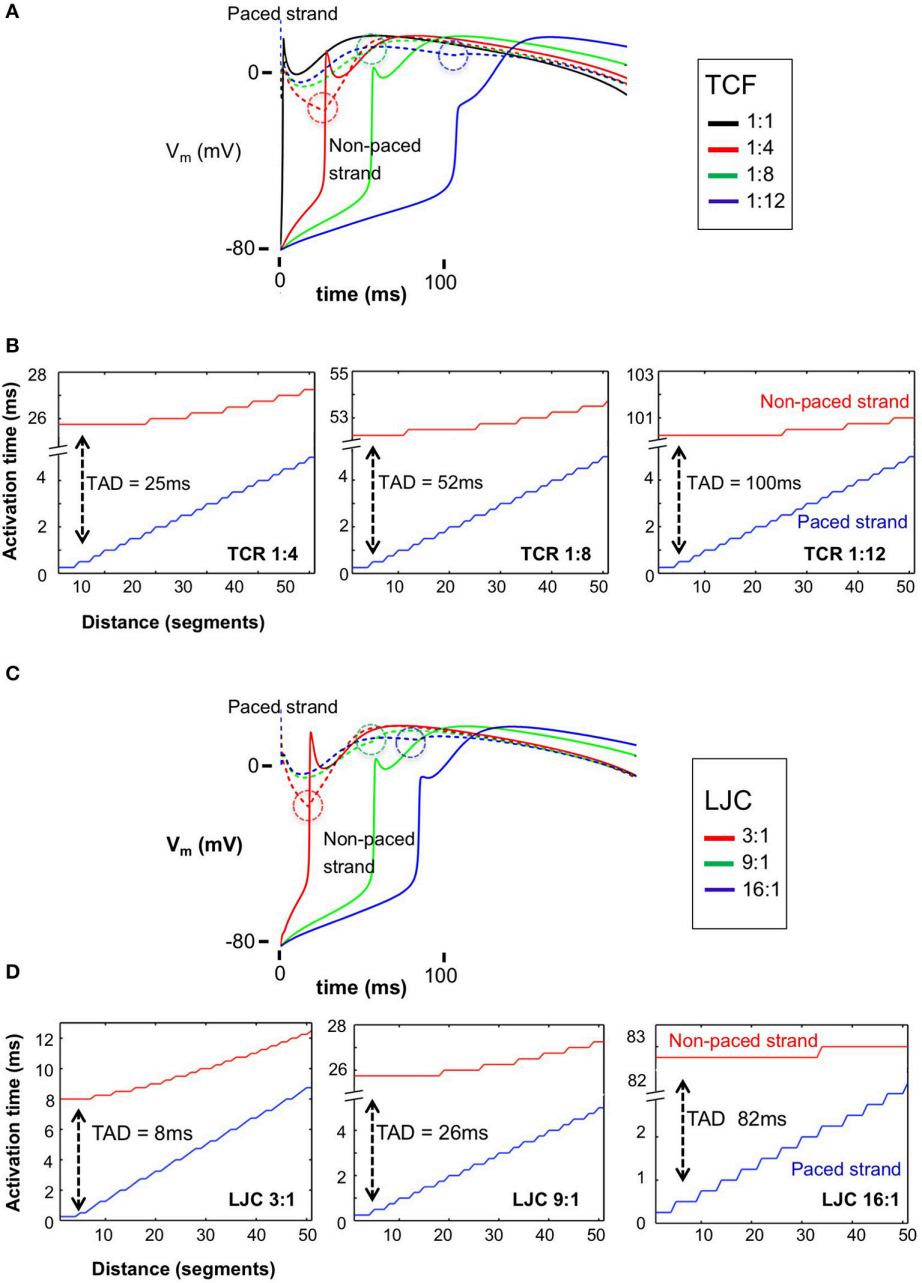
Action potentials (APs) and transverse activation delay (TAD) for the paced and non-paced strands with varied discrete TCF and LJC. **(A)** APs for the stimulus location in the paced strand (dashed lines) and the directly opposite myocyte in the non-paced strand (solid lines) with TCF = 1:1, 1:4, 1:8, 1:12, respectively. The dashed circles highlight the timing of electrotonic interaction between the paced and non-paced strands; once the non-paced strand was activated, it acted as a current source and that elevated the potential in the paced strand. **(B)** TADs increased dramatically with decreased TCF from 25 ms (TCF = 1:4) to 100 ms (TCF = 1:12), while having little impact on conduction velocity in the paced strand. On the other hand, after a long TAD, activation of the non-paced strand is almost instantaneous. **(C)** APs for the stimulus location in the paced strand (dashed lines) and the directly opposite myocyte in the non-paced strand (solid lines) with LJC = 3, 9 and 16:1, respectively. The dashed circles highlight the timing of electrotonic interaction between the paced and non-paced strands. **(D)** TADs increased dramatically with increased LJC from 8 ms (LJC = 3) to 82 ms (LJC = 16), while having little impact on conduction velocity in the paced strand. With a higher LJC, and a longer TAD, the degree of synchronicity of activation within the non-paced strand increased.

**Figure 3:**
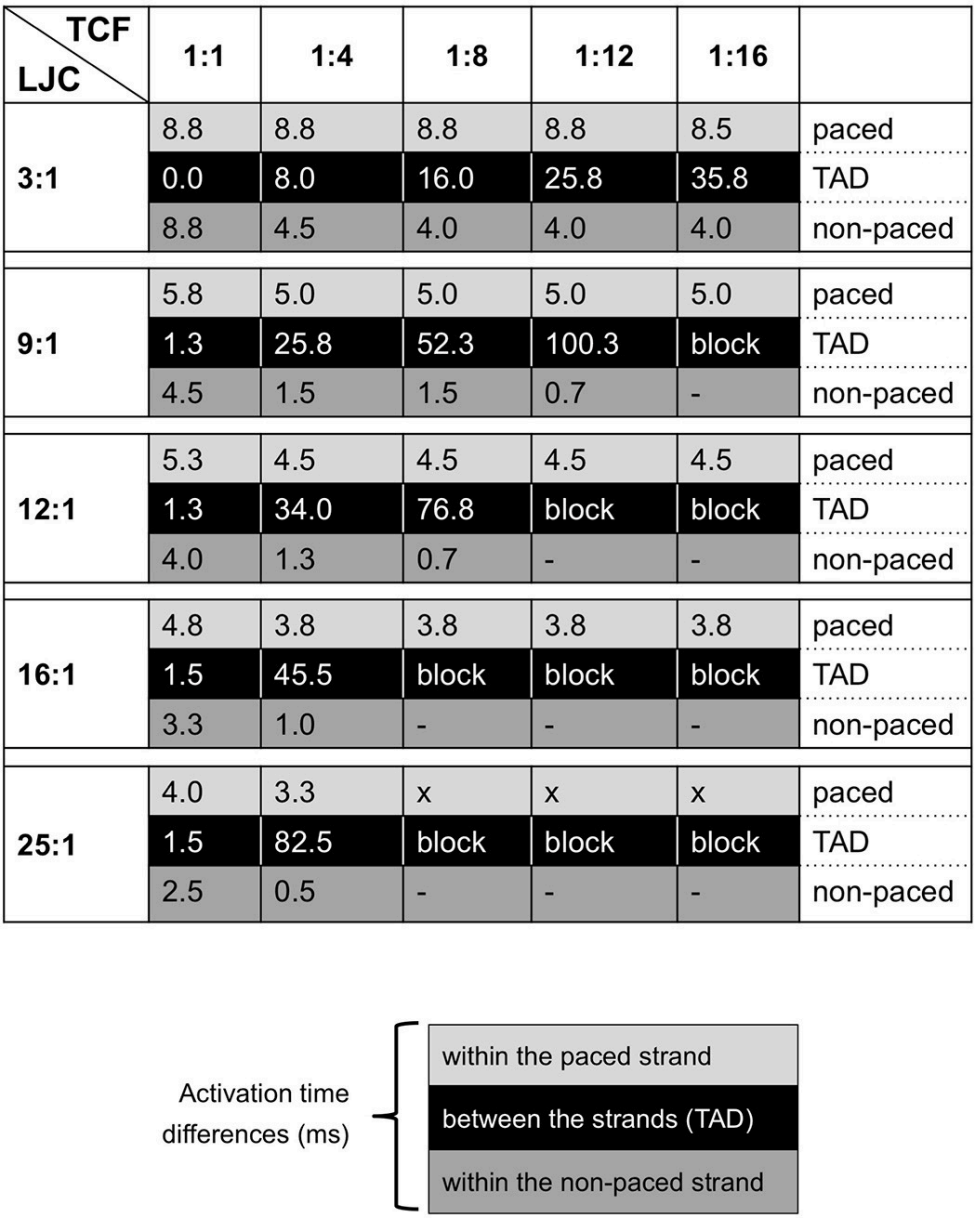
Summarizing table for TAD and activation delays (in milliseconds) in both strands with varied TCF and LJC. Black blocks indicate the TAD; gray and light gray blocks contain entries for activation delays for the non-paced and paced strands, respectively.

When the transverse connectivity fraction (TCF) was reduced from 1:1 to 1:4, 1:8, 1:12, and 1:16, the conduction velocity (CV) in the stimulated strand was not affected (~1 mm/ms; blue lines in Figure 2B). However, the shape of the action potential plateau in the electrically paced strand was affected by electrotonic interaction with the non-paced strand, which acted as a current sink before its moment of activation. Once the non-paced strand was activated, it acted as a current source and that elevated the potential in the paced strand (circled deflections in Figure 2A). With decreasing TCF, the delay between the earliest activation in the paced and non-paced strands dramatically increased (Figures 2B, 3). After this prolonged activation delay between the two strands, the activation propagated more rapidly in the non-paced strand than in the paced strand. Interestingly, the propagation within the non-paced strand became faster with a progressive loss of transverse connections, leading to a virtually simultaneous activation of the non-paced strand with a TCF of 1:12 (Figures 2A,B). A further reduction in TCF to 1:16 lead to transverse conduction block (not shown).

### Effects of axial impedance on transverse propagation

When depolarizing current flows from the paced to the non-paced strand through a lateral connection, part of that current quickly dissipates in the axial direction within the non-paced strand. To investigate the strength of this effect, we increased the longitudinal junctional conductance (LJC), while keeping the transverse junctional conductance and transverse connectivity fraction unchanged. This corresponded to an anisotropy ratio (longitudinal vs. transverse conductance) varying from 3:1 to 25:1. Over this range, we observed an increase in CV in the paced strand as expected, as well as an increasing transverse activation delay (Figures 2C,D, 3). With a TCF of 1:4 and LJC 3:1, the average CV in the paced strand was 0.57 m/s, increasing 1.7 and 2.9-fold at an LJC of 9:1 and 25:1, respectively. Increasing the LJC caused a pronounced increase in transverse conduction delays, e.g., 8, 26, and 82 ms for LJC of 3:1, 9:1, and 25:1, respectively. After the transverse activation delay, the activation of the non-paced strand was more synchronous than that of the stimulated strand. The synchronicity of activation was enhanced when the TCF was decreased and especially when the axial conductance was increased as well.

### Effects of sodium and calcium channel blockade

**Figure 4:**
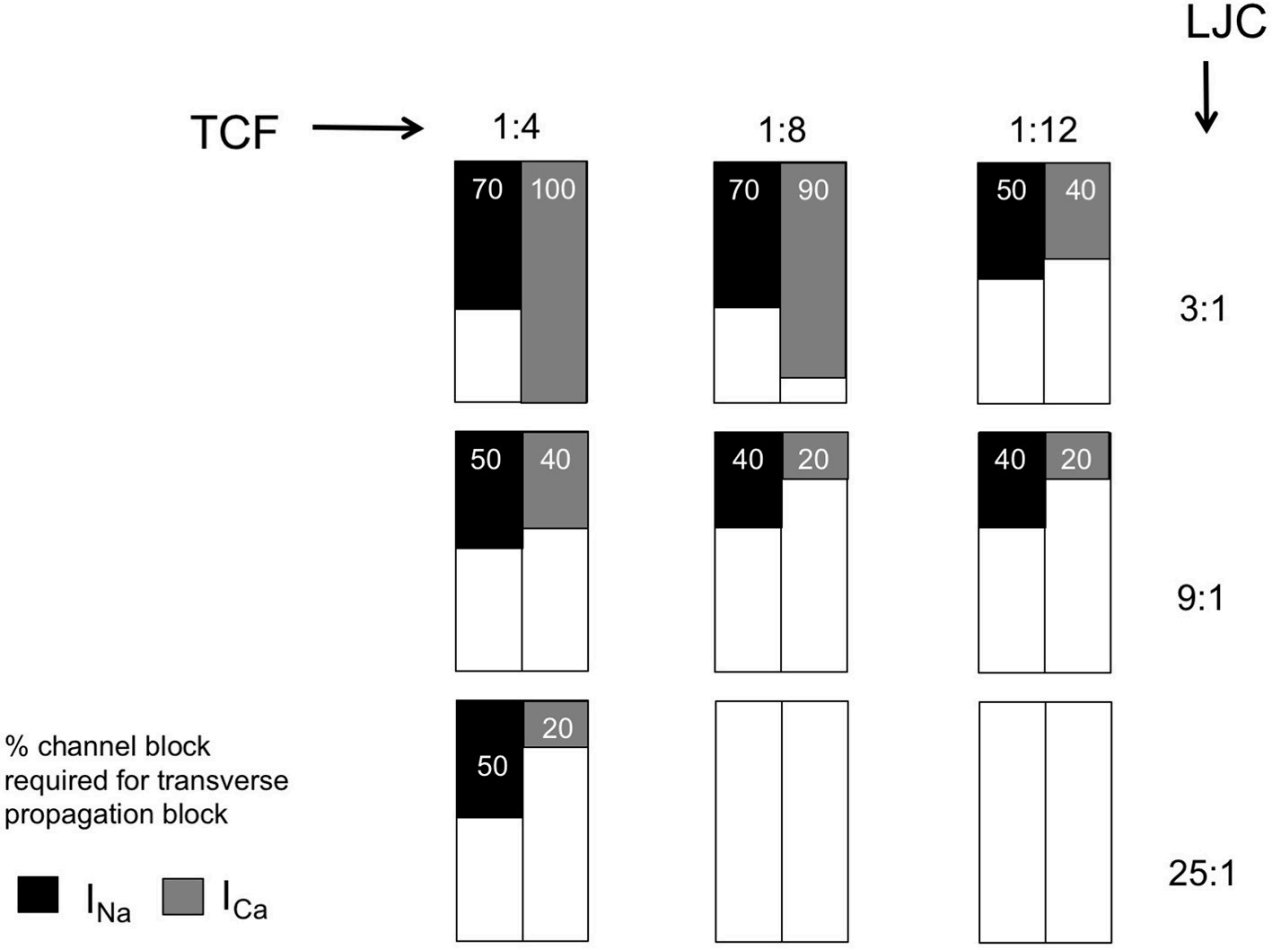
Sensitivity of transverse propagation to sodium and calcium current (I_Na_ and I_Ca_) block. Summarizing table with entries representing the minimal percentage of channel block required for transverse block with varied discrete TCF and LJC.

To assess the effects of G_Na_ and G_Ca,L_ channel blockers, a stepwise reduction of g_Na_ and g_Ca,L_ conductance in the computer model was implemented until transverse conduction block was observed (Figure 4). With high transverse coupling (1:1 or 1:4) and relatively low LJC (3:1), transverse propagation was relatively more sensitive to sodium current blockade. Under these conditions, transverse propagation could be blocked by 70% g_Na_ blockade, but still occurred with 100% g_Ca,L_ blockade. Increasing axial conductance dramatically increased the sensitivity to calcium channel blockade, with transverse block occurring at 40 and 20% calcium channel blockade at an LJC of 9:1 and 25:1. Similarly, decreasing the TCF strongly enhanced the sensitivity to calcium channel blockade, with transverse block observed at 90 and 40% with a TCF of 1:8 and 1:12, respectively. Qualitatively, simulations with sodium channel blockade showed similar trends, but the sensitivity to changes in TCF and LJC was less pronounced. Whereas transverse propagation still occurred with 100% G_Ca,L_ blockade at a TCF of 1:4 and a LJC of 3:1, transverse propagation failed at 40% G_Ca,L_ blockade when TCF was lowered to 1:12 and at 20% G_Ca_ blockade when LJC was increased to 25:1. Under the same conditions, transverse propagation failed at 70, 50, and 50% G_Na_ blockade, respectively (Figure 4). For the combination of LJC = 25:1 and low TCF (1:8 or 1:12), transverse propagation failure was always observed, even in the absence of sodium or calcium channel blockade.

These results demonstrate that both sodium and calcium current contribute to transverse propagation, because both sodium and calcium channel block can cause failure of transverse propagation.

### The roles of sodium and calcium in transverse propagation

**Figure 5:**
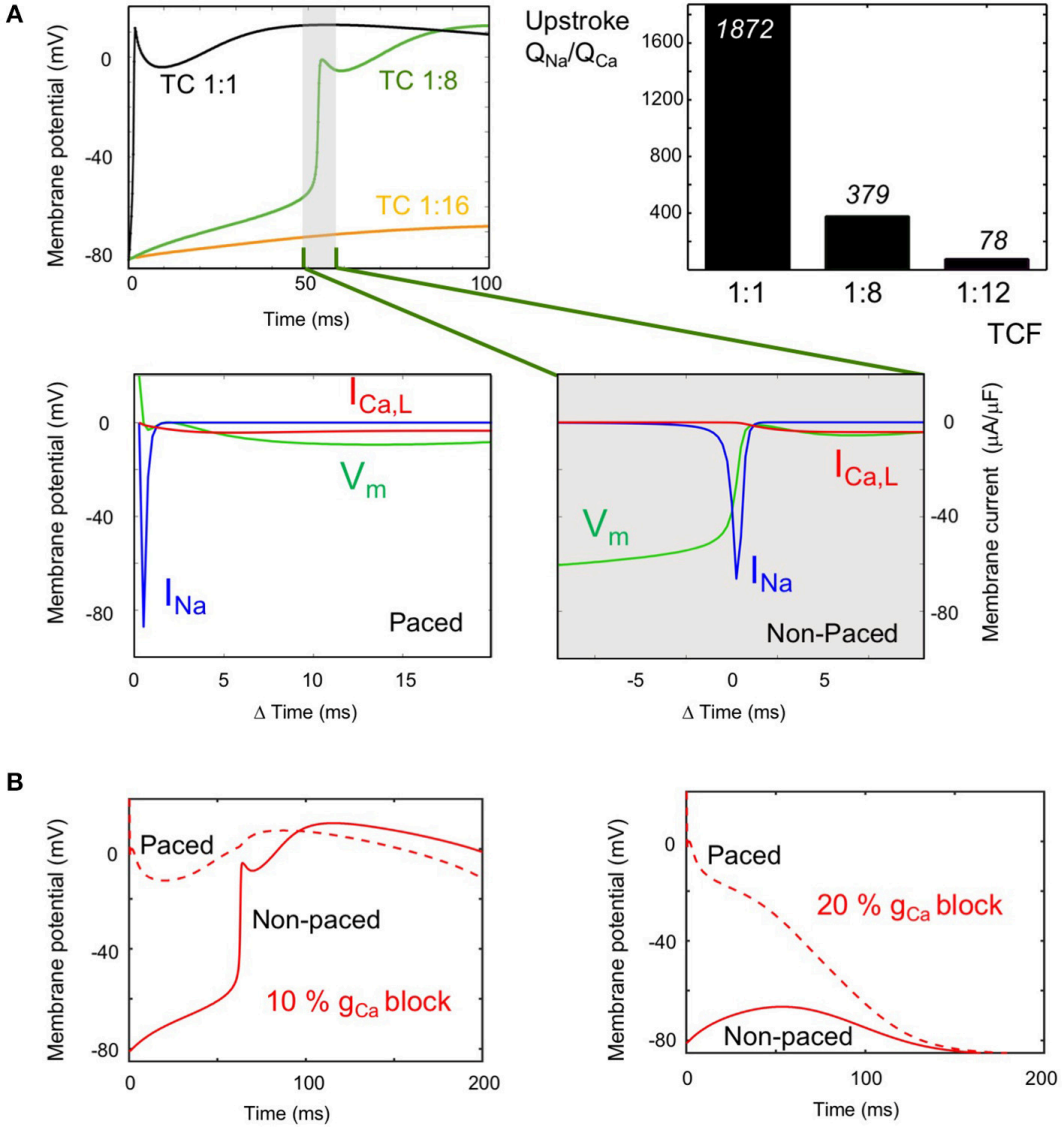
Role of the sodium and calcium current (I_Na_ and I_Ca_) in transverse propagation. **(A)** I_Na_ and I_Ca_ during the upstroke at long TADs (LJC = 9:1). Top left: Action potentials (APs) in the non-paced strand, at the site directly opposing the pacing site, with TCF 1:1 (black), 1:8 (long TAD, green), and 1:16 (propagation failure, orange). Bottom row: V_m_ (green), I_Na_ (blue) and I_Ca_ (red) during upstroke of the AP at a TCF of 1:8 are plotted for the paced strand (left panel) and the non-paced strand (right panel). The magnitude of I_Na_ during the upstroke in the non-paced strand is several hundred times of that of I_Ca_. Top right panel: Calculated ratio of charge carried by the sodium and calcium current during the AP upstroke in the non-paced strand at various TCFs (LJC = 9:1). **(B)** Role of I_Ca_ in transverse propagation (LJC = 9:1, TCF 1:8). APs are displayed for the paced strand (dashed line) and non-paced strand (solid line) for 10% I_CaL_ blockade, where transverse propagation was successful (left panel) and for 20% I_CaL_ blockade, where transverse conduction block occurred (right panel).

The role of the sodium and calcium currents in the activation of both strands was distinctly different. With an LJC of 9:1 and a TCF of 1:8, there was a long transverse conduction delay of 52 ms (top left panel in Figure 5A). However, even after this long delay, the action potential upstroke in the non-paced strand is still dependent on the sodium current (bottom left panel in Figure 5A). At the moment of activation of the non-paced strand, the sodium current in the paced strand had already completely inactivated. Therefore, the calcium current maintaining the action potential plateau in the paced strand was essential in providing sufficient depolarizing current for the sodium-dependent upstroke to occur in the non-paced strand. In Figure 5A, the right panel plots the ratio of the amount of depolarizing charge carried by the sodium and calcium currents during the AP upstroke in the non-paced strand. Although this ratio Q_Na_/Q_Ca_ strongly decreases when the TCF is reduced, the preponderance of the charge is still carried by the sodium current under all conditions in which transverse propagation occurs.

Figure 5B compares the action potential morphologies (LJC = 9:1 and TCF = 1:8) at 10% G_Ca,L_ blockade, which still allows transverse propagation and 20% G_Ca,L_ blockade, which leads to transverse propagation failure. When I_Ca,L_ in the paced strand is sufficient to support transverse propagation, the AP in the paced strand is prolonged by the electrotonic influence of the activated non-paced strand (left panel). When transverse propagation fails at a slightly higher degree of G_Ca,L_ blockade, the AP in the paced is markedly shorter (right panel).

## Discussion

Propagation of the activation wavefront in cardiac tissue primarily depends on the I_Na_ current density and the degree of gap junctional coupling. In a seminal study, Shaw and Rudy have used a mathematical model of a single strand of myocytes to compare the effects of reductions in these two factors (Shaw and Rudy, 1997). They demonstrated that I_Na_ blockade conduction velocity decreased from 54 to 17 cm/s before conduction block occurred. However, when gap junctional coupling was reduced, a much lower conduction velocity of 0.26 cm/s was reached before conduction blocked. With strongly reduced gap junctional coupling, propagation became almost entirely dependent on I_CaL_. This modeling study agrees with an earlier study in patterned cultures of neonatal myocytes, where a thin strip of myocytes widened into a broader monolayer (Rohr et al., 1997). Whereas conduction normally blocked at the widening point, the thin strip was able to excite the broader area during partial gap junctional uncoupling with octanol, illustrating that an increase in gap junctional resistance can paradoxically prevent conduction block.

In the normal adult myocardium, neighboring myocytes are well coupled at their end-to-end (longitudinal) connections (Desplantez et al., 2007). However, the distribution of transverse connections is more discrete and sparse, both between individual myocytes and between neighboring bundles. This sparsity of lateral connections differs between regions in the healthy heart (Dolber and Spach, 1987). For example, in Bachmann's bundle, the distance between lateral connections between funicles (sub-bundles) may amount to 2 mm (Dolber and Spach, 1989). The development of endomysial/ interstitial fibrosis specifically affects transverse connectivity, probably by increasing the sparsity of lateral connections between myocytes and myocyte bundles. This process occurs during normal aging (Spach and Dolber, 1986; Koura et al., 2002; Spach et al., 2007) and is exacerbated by structural remodeling that occurs during AF. In a goat model, 6 months of AF caused endomysial fibrosis specifically in the thin epicardial layer of the atrial wall, selectively impairing transverse propagation of epicardial wavefronts (Verheule et al., 2013, 2014).

The central question of this study is how the sparse nature of lateral connections affects cardiac propagation and the relative contributions of the sodium and calcium currents to propagation. The main findings of our study can be summarized as follows, as discussed sequentially below:

1. Reducing transverse coupling increases the transverse activation delay; this is exacerbated when axial conductivity is high.
2. With reduced transverse coupling and increased axial conductivity, the spread of activation within the non-paced strand becomes faster.
3. Even after a long transverse activation delay, the action potential upstroke in the non-paced strand is still I_Na_ -dependent, but I_Ca_ is essential for transverse propagation by sustaining the plateau in the paced strand.
4. Increased axial conductivity or decreased transverse connectivity increases sensitivity to I_Ca_ block to a greater extent than the sensitivity to I_Na_ block.

### Transverse activation delay (ad 1)

In our simulations, the TAD was highly sensitive to both the TCF and the LJC. When the TCF was decreased and the LJC was increased, the paced strand was completely activated before transverse propagation occurred. Thus, a loss of transverse connections precipitates discontinuous transverse propagation. As a starting condition, we used a TCF of 1:1, i.e., all units in the parallel strands were coupled transversely. Under this condition, the TAD was small at all LJCs investigated. However, even in the healthy adult heart, the degree of transverse connectivity is probably much lower, especially in regions with a strong preferential fiber orientation such as Bachmann's bundle (Dolber and Spach, 1987, 1989). This normal sparsity of transverse connections between bundles would increase the propensity to longer TADs, and it would increase further when fibrosis leads to a loss of transverse connections.

We varied the LJC to assess to which extent transverse propagation is affected by a lower axial impedance. In theory, depolarizing current flowing through a transverse connection would more readily dissipate in the axial direction of the non-paced strand with a higher LJC. This would make it more difficult to reach the activation threshold of the non-paced strand, leading to a longer TAD or even transverse block. Indeed, our simulations show that the TAD is very sensitive to the LJC, increasing from 8 ms at an LJC of 3:1 to 83 ms at an LCJ of 25:1 (TCF 1:4). With a further reduction in TCF, an increase in LJC quickly led to block of transverse propagation. In a single strand of myocytes, a “safety factor” for conduction can be calculated as the ratio between the charge generated by a cell upstream in the strand and the charge required to bring the next cell to threshold (Shaw and Rudy, 1997). In our model however, the charge generated by an activated myocyte in the paced strand not only flows to a connected myocyte in the non-paced strand, but to a larger extent to myocytes in the axial direction, precluding a straightforward determination of a safety factor. With an increase in axial conductivity and a decrease in transverse connectivity, the paced strand activates first, with the entire strand contributing to depolarization of the non-paced strand. In several experimental models, structural remodeling, and the concomitant loss of side-to-side connections and cellular hypertrophy primarily affects transverse propagation, with minor effects on longitudinal propagation (Stein et al., 2008; Glukhov et al., 2012). Based on cable theory, an increase in cell diameter should decrease axial impedance and increase longitudinal conduction velocity, as has indeed been shown for hypertrophic ventricular myocardium in a rabbit model of heart failure (Wiegerinck et al., 2006). Structural remodeling caused by AF is characterized both by endomysial fibrosis and myocyte hypertrophy (Ausma et al., 2003; Verheule et al., 2013). These two factors may thus act synergistically to produce discontinuous transverse conduction and transverse conduction block.

### Activation pattern within the strands (ad 2)

As expected, activation of the paced strand became slightly faster with increasing LJC (activation time decreased from 8.8 ms at LJC 3:1 to 4 ms at LJC 25:1). By contrast, the activation of the paced strand was relatively insensitive to the TCF, indicating that current leak through transverse connections did not significantly affect longitudinal conduction in our model. As noted above, an increase in LJC and decrease in TCF increased the TAD. However, after this longer TAD, activation within the non-paced strand became faster (activation time 8.8 ms at TCF 1:1 and LJC 3:1 vs. 0.7 ms at TCF 1:8 and LJC 12:1, for example). With a long TAD, the paced strand has already activated completely, allowing depolarizing current to flow through numerous adjacent transverse connections, jointly bringing the non-paced strand to its activation threshold. Especially at high LJC and low TCF, the activation within the non-paced strand is essential synchronous. For this reason, a conduction velocity cannot be calculated for the non-paced strand. In effect, this near-instantaneous activation (after a long delay) translates to a 90-degree turn in propagation direction between the two simulated strands, from longitudinal in the paced strand to transverse in the non-paced strand. A loss of transverse connections may thus paradoxically precipitate discontinuous transverse propagation. Depending on local fiber architecture, this phenomenon would also promote sharp changes in propagation direction and may therefore in part explain experimental observations of zig-zag conduction (in canine atria as a result of aging) (Koura et al., 2002) and microreentry (in human atria) (Hansen et al., 2015).

### Contribution of I_Na_ and I_Ca_ (ad 3)

In simulations on a single strand, the upstroke of the action potential became increasingly dependent on I_Ca_ when gap junctional conductance was reduced (Shaw and Rudy, 1997). The long TAC that occurs in our double strand model when LJC was increased and/ or TCF was decreased entails that I_Na_ had already completely inactivated when the non-paced strand was activated, and could therefore not supply depolarizing current over transverse connections at that point in time. Instead, I_Ca_ was responsible for maintaining the action potential plateau of the paced strand long enough for transverse propagation to occur. Nevertheless, even with long TADs, the majority of the current during the action potential upstroke of the non-paced strand was still carried by I_Na_.

### Sensitivity to channel block (ad 4)

The diverging roles of I_Na_ and I_Ca_ described above translate into different sensitivities to channel block under the conditions we have tested. Overall, the degree of I_Na_ block required to block transverse propagation decreased both when TCF was decreased and when LCJ was increased. However, the degree of I_Ca_ block required to block transverse propagation was much more sensitive to both parameters. At a TCF of 1:4 and an LJC of 3:1, transverse propagation still occurred with 100% I_Ca_ block, but 40% I_Ca_ block was sufficient to prevent transverse propagation with a TCF of 1:12 and 20% I_Ca_ block prevented transverse propagation with an LJC of 25:1. The dependence on TCF implies that sensitivity to I_Ca_ block increases strongly with a loss of transverse connections, i.e., during structural remodeling. Anti-arrhythmic drugs can be used for cardioversion at early stages in the pathogenesis of AF both in AF patients (Crijns et al., 1988) and in animal models, but their efficacy declines when structural remodeling develops (Eijsbouts et al., 2006; Verheule et al., 2010). Fibrillatory conduction in structurally remodeled atria is characterized by dissociated propagation patterns that are consistent with loss of transverse connectivity (Verheule et al., 2013). According to our model and earlier experimental and modeling studies this loss of transverse connectivity would favor discontinuous transverse propagation (Spach and Dolber, 1986; Spach and Boineau, 1997; Christensen et al., 2015). As an anti-arrhythmic strategy, I_Ca_ blockade has not been successful,(Villani et al., 2000; Manios et al., 2003; Hemels et al., 2006) possibly because potential anti-arrhythmic effects (prevention of calcium overload and discontinuous conduction) are offset by the pro-arrhythmic effect of action potential shortening. However, because of the different roles of I_Na_ and I_Ca_ in transverse propagation, a potential strategy would be combined treatment with an I_Ca_ blocker and an I_Na_ or I_K_ blocker. To our knowledge, this approach has not been investigated either in animal models of AF or in AF patients.

## Limitations

Our “proof-of principle” model consists of two 3-dimensional strands. These strands correspond to small bundles or “funicles,” which have been demonstrated to be important determinants of microscopic conduction (Dolber and Spach, 1987, 1989). The model displayed long time delays in transverse propagation. We do not know to what extent the ranges in our simulations exceed the pathophysiological range, but discontinuous transverse propagation in the atria has been observed in isolated bundles and intact hearts (Spach et al., 1982; Verheule et al., 2010). It is likely that with more parallel bundles, TADs would become smaller, because more transverse connections can deliver depolarizing current from bundles that have already been activated to a bundle that is still at rest. However, we believe the same dependencies of transverse propagation on the transverse connectivity and axial conductance would still hold. In addition, TADs would become larger, also in 3-dimensional structures, during premature activations or AF, (Spach et al., 2007) when I_Na_ and I_Ca_ cannot fully recover from inactivation, and this mechanism could thereby contribute to the dissociated fibrillation patterns observed in structurally remodeled atria (Verheule et al., 2010).

Several mathematical models are available for atrial cellular electrophysiology. We have used the Courtemanche model for a human atrial myocyte (Shaw and Rudy, 1997), a widely used and generally accepted model. However, we do not expect our findings to be model-dependent. The relative contributions of the sodium and calcium current in transverse propagation depend mainly on their current density and kinetics, which are quite similar between models. This does not mean that alterations in cellular electrophysiology will not have an effect. For example, AF-induced electrical remodeling shortens the APD. We expect that this would increase the propensity for transverse propagation failure, and failure would then occur already at a higher degree of transverse coupling. However, the nature of the dependencies on TCF and LJC would remain the same.

The study by Shaw and Rudy simulated the effects of LJC in a single, one-cell wide, strand of ventricular myocytes (Shaw and Rudy, 1997). Here, we have simulated thin 3-dimensional strands of atrial myocytes, because we believe that this is more pertinent to the tissue architecture of the atria. Although our study cannot be directly compared to the study by Shaw and Rudy in all respects, we propose that the sparse nature of transverse connection is an important determinant of cardiac propagation and that contribution and I_Na_ and I_Ca_ differs between longitudinal and transverse propagation.

## Author contributions

JZ worked on computer simulations, data analysis, and writing. US and BS critically evaluated the manuscript. SV ensured the overall coordination of the article, and worked on data analysis and writing.

### Conflict of interest statement

The authors declare that the research was conducted in the absence of any commercial or financial relationships that could be construed as a potential conflict of interest.
